# Antiplatelet Therapy of Cilostazol or Sarpogrelate with Aspirin and Clopidogrel after Percutaneous Coronary Intervention: A Retrospective Cohort Study Using the Korean National Health Insurance Claim Database

**DOI:** 10.1371/journal.pone.0150475

**Published:** 2016-03-03

**Authors:** Yoojin Noh, Jimin Lee, Sooyoung Shin, Hong-Seok Lim, Soo Kyung Bae, Euichul Oh, Grace Juyun Kim, Ju Han Kim, Sukhyang Lee

**Affiliations:** 1 College of Pharmacy, Ajou University, Suwon, South Korea; 2 Department of Cardiology, School of Medicine, Ajou University, Suwon, South Korea; 3 College of Pharmacy, The Catholic University of Korea, Bucheon, South Korea; 4 Division of Biomedical Informatics, College of Medicine, Seoul National University, Seoul, South Korea; University Hospital Medical Centre, GERMANY

## Abstract

**Background/Objectives:**

Addition of cilostazol or sarpogrelate to the standard dual antiplatelet therapy of aspirin and clopidogrel has been implemented in patients that underwent percutaneous coronary intervention (PCI) with stents in Korea. This study aimed to evaluate the efficacy and safety of triple antiplatelet therapies.

**Methods:**

This retrospective cohort study was performed using the Korean National Insurance Claim Data of the Health Insurance Review and Assessment Service from January 1, 2009 to December 31, 2014. The study cohort population consisted of patients with ischemic heart diseases and a history of PCI. They were treated with antiplatelet therapy of aspirin, clopidogrel (AC); aspirin, clopidogrel, cilostazol (ACCi); or aspirin, clopidogrel, sarpogrelate (ACSa) during the index period from January 1, 2010 to December 31, 2011. During the follow-up period up to December 31, 2014, the major adverse cardiac or cerebral events (MACCE) including death, myocardial infarction, target lesion revascularization, and ischemic stroke were assessed. Bleeding complications were also evaluated as adverse drug events.

**Results:**

Out of 93,876 patients with PCI during the index period, 69,491 patients started dual (AC) or triple therapy (ACSa or ACCi). The clinical outcomes of comparing ACSa and ACCi therapy showed beneficial effects in the ACSa group in the prevention of subsequent cardiac or cerebral events. After Propensity score-matching between ACSa and ACCi groups, there were significant differences in MI and revascularization, with corresponding HR of 0.38 (95% CI, 0.20–0.73) and 0.66 (95% CI, 0.53–0.82) in ACSa vs. ACCi at 12 months, respectively. At the 24-month follow-up, the triple therapy groups (ACS or ACC) had a higher incidence of MACCE compared to the dual therapy (AC) group; ACSa vs. AC HR of 1.69 (95% CI, 1.62–1.77); ACC vs. AC HR of 1.22 (95% CI, 1.06–1.41). There was no significant difference in severe or life-threatening bleeding risk among three groups; ACSa vs. AC, HR of 0.68 (95% CI, 0.37–1.24), ACCi vs. AC, HR of 0.91 (95% CI, 0.77–1.09).

**Conclusion:**

Sarpogrelate-containing triple antiplatelet therapy demonstrated comparable rates of MACCE prevention to the conventional dual antiplatelet therapy after PCI without significantly increasing bleeding risk during the two-year follow-up period.

## Introduction

Dual antiplatelet therapy consisting of aspirin and P2Y12-receptor antagonist, especially clopidogrel, prasugrel or ticagrelor is currently recommended for prevention of cardiovascular events in clinical guidelines as standard therapy for patients undergoing percutaneous coronary intervention (PCI) with coronary stent [1–3]. However, treatment failure has occurred due to heterogeneity in the response of individual patients to aspirin or clopidogrel [4]. Resistance to aspirin or clopidogrel has been seen clinically, and there is a relatively high prevalence of clopidogrel resistance in Asia [5–7].

Triple antiplatelet therapy consisting of aspirin, clopidogrel, and cilostazol has been suggested as an effective measure to addresses the possibility of treatment failure due to resistance. The increased risk of bleeding over the standard dual drug therapy could be a safety concern, but safety as well as efficacy of triple therapy with cilostazol had been studied and reported previously [8–13]. The guideline of antiplatelet therapy in Korea also recommends cilostazol as a triple antiplatelet therapy to overcome resistance of clopidogrel [14].

Sarpogrelate is another potential agent for adjunctive antiplatelet therapy, and has been approved in Japan since 1993. Sarpogrelate is a selective 5-hydroxytryptamine receptor subtype 2A antagonist, which acts as a platelet aggregation inhibitor to improve peripheral circulation in the treatment of ischemic symptoms observed in patients with chronic arterial obstruction [15]. Studies have reported on the effectiveness of sarpogrelate in patients with ischemic heart disease or peripheral vascular disease [16, 17]; however, most of the studies were small clinical trials investigated in the Asian population. Data on its efficacy as part of triple antiplatelet therapy (aspirin, clopidogrel, and sarpogrelate) in patients undergoing stent are scarce [18, 19]. Considering Asian individual characteristics, it is necessary to evaluate the efficacy and safety of triple therapy with sarpogrelate or cilostazol after PCI with coronary stent, because no studies have compared the efficacy and safety of the two triple therapy combinations. The purpose of this study is to evaluate the treatment pattern and effect in large-scale healthcare claim data of two triple antiplatelet therapy options commonly used in Korea after stent implantation.

## Methods

### Study Design and Population

The retrospective cohort study was conducted to evaluate the efficacy and safety of antiplatelet therapies after percutaneous coronary intervention (PCI) with stent implantation. This study used the administrative data from the Health Insurance Review and Assessment Service (HIRA) database in Korea. The HIRA database includes the demographic information and medical benefit claim information of approximately 50 million Korean people. The HIRA database includes data on all ambulatory and inpatient claims regarding ambulatory care service, inpatient orders, and prescriptions dispensed at pharmacies. The national health insurance (NHI) program is a universal health care system, requiring a contribution of a monthly premium and a copayment, which allows beneficiaries to access any of the contracted medical facilities and institutions in Korea. The majority of Korean people are represented in the NHI claims database.

To protect patient privacy, personal identification numbers were codified or blocked. The authors were blinded to the personal identification numbers. The diagnoses were coded according to the Korean Classification of Disease (KCD-6) modification of the International Classification of Disease (ICD-10). The clinical procedures were coded by the HIRA coding system. Information for prescribed drugs included brand name, generic name, dose, prescription date, and duration of therapy.

**Fig 1:**
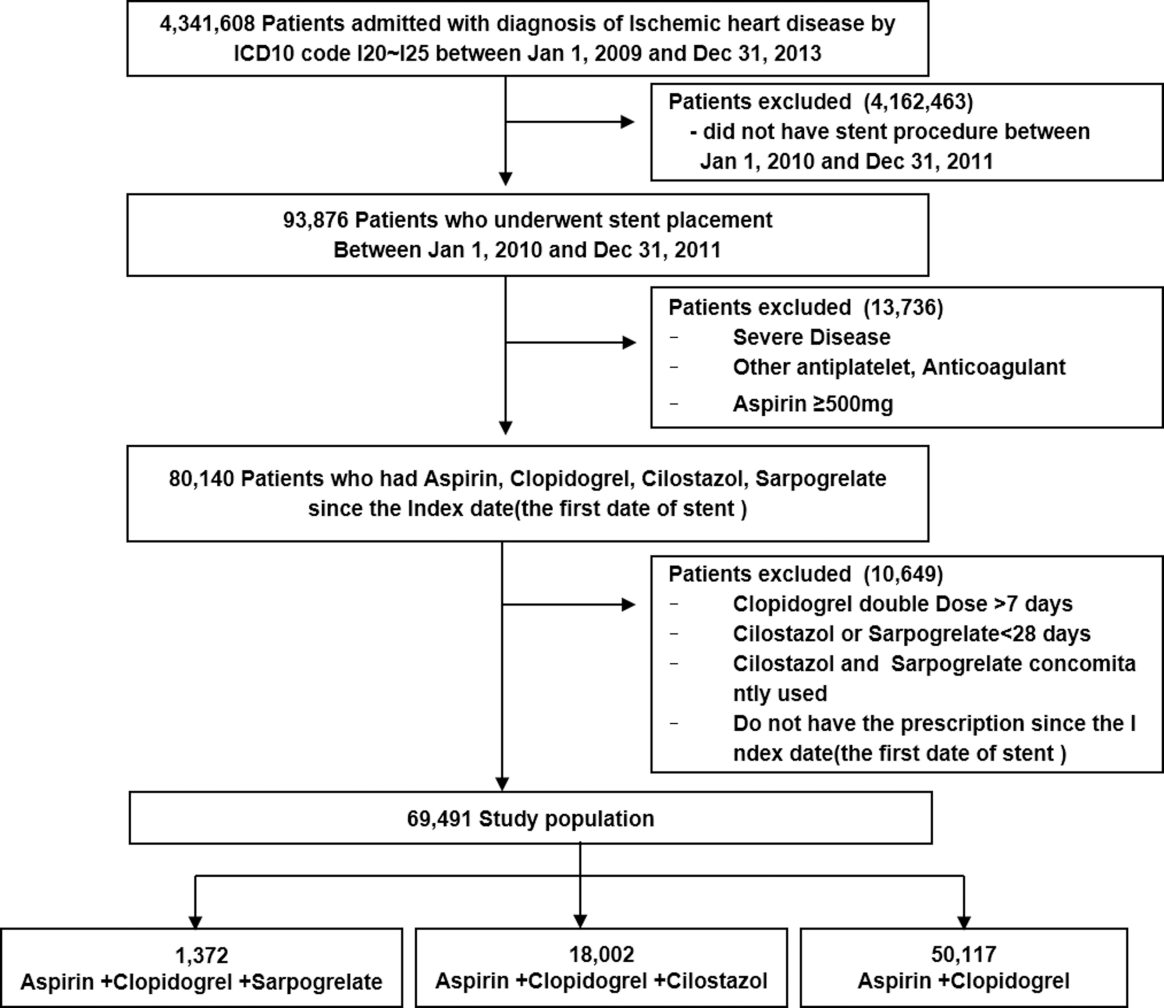
Flow chart of the study population.

We obtained the claim information for patients who were diagnosed with ischemic heart disease (ICD-10 codes I21-I25) between January 1, 2009 and December 31, 2013. The study population undergoing stent from January 1, 2010 to December 31, 2011 was collected out of the claim database for patients who were diagnosed with the ischemic heart disease during the relevant period. The coronary stent classification codes used were “Percutaneous Transcatheter Placement of Intracoronary Stent-Single Vessel” and “Percutaneous Transcatheter Placement of Intracoronary Stent-Additional Vessel”. The index date was defined as the first date of stent procedure during the study period (Fig 1). Inclusion criteria were patients treated with the target antiplatelet agents for 12 months: aspirin and clopidogrel, with or without cilostazol or sarpogrelate as a third agent. Eligible patients were classified into one of three study groups: dual therapy group (aspirin + clopidogrel, AC); triple therapy group (aspirin + clopidogrel + cilostazol, ACCi); or triple therapy group (aspirin + clopidogrel + sarpogrelate, ACSa). Current guidelines recommend the duration of antiplatelet therapies up to 12 months depending on the patients’ characteristics [1–3]. Patients was treated with the study therapy for 12 months, and considered continuously exposed to a study therapy until patients discontinued their therapy or switched to the other antiplatelet therapy. The censored time was calculated for the treatment duration.

Patients treated with other antiplatelet agents from the index date were excluded. Exclusion criteria also included the following: (1) patients with severe liver disease (ICD-10 code K72.1), chronic renal disease (stage 4 and 5, ICD-10 codes N18.4, N18.5), and heart failure (ICD-10 codes I50), (2) under 18 years of age, (3) patients treated with other antiplatelet and anticoagulants such as warfarin, GP IIb/IIIa, vitamin K antagonist and factor Xa inhibitors (4) patients who had over 500 mg of aspirin or clopidogrel double dose over 7 days, (5) patients treated with cilostazol or sarpogrelate as a triple therapy for fewer than 28 days, (6) patients who received both cilostazol and sarpogrelate concomitantly for the study period, and (7) patients who took the study drug therapy for 6 months or less.

### Study Endpoint, Definitions, and Follow-Up

The primary efficacy endpoint was a major adverse cardiac or cerebral event (MACCE), which was defined as one of the following: all-cause death (ICD-10 codes R96, R98, R99, and I46.1) [20], ischemic stroke (ICD-10 codes I63, I64), recurrent MI (ICD-10 codes I21, I22), or revascularization. The safety assessment was severe or life-threatening or minor bleeding event at 12 months. Severe or life-threatening bleeding was defined as subarachnoid, intra-cerebral, or intracranial hemorrhage. Bleeding definition was based on the GUSTO criteria [21], but it was determined by ICD-10 code which was an available data in this study. MI was determined after the discharge dates. To ensure only appropriate inclusion of myocardial infarction event as a recurrence, both the second MI diagnosis code and a revascularization code should occur within the same period. Revascularization was determined as a stent procedure code or bypass surgery, occurring more than 7 days from the index date to exclude pre-planned procedures. Ischemic stroke as the primary diagnosis should be recorded more than twice in order to be a qualifying diagnosis. The patients were followed up to 24 months from the index date, and all of the endpoints were measured at 12 months and 24 months. For determining impact of long term period therapies, the maximum follow up period was defined 24 months [11, 22].

Clinical outcomes of antiplatelet therapies were assessed according to the characteristics of the patient, including age at index date, gender, comorbidities, and concomitant medications. Comorbidities included hypertension, cerebrovascular disease, peripheral arterial disease, hyperlipidemia, and diabetes mellitus. The appropriate ICD-10 codes must be recorded more than twice in the 1 year prior to the index date to qualify as comorbidity. The Charlson comorbidity score was calculated in the year prior to the index date [23]. Concomitant medications considered were angiotensin converting enzyme inhibitors, angiotensin receptor blockers, β-blockers, calcium channel blockers, nitrates, statins, other lipid lowering agents, and proton pump inhibitors between 60 days before and 60 days after the index date. These medications were not included as concomitant medications if prescribed only one time for a period of less than 7 days.

### Statistical Analysis

Baseline characteristics and incidence of events are presented as numbers with percentage for categorical variables. Continuous variables expressed as mean and standard deviation were compared using ANOVA statistical analysis. Categorical variables were compared with the Mantel–Haenszel chi-squared test. The patient characteristics were also compared by standardized mean difference [24]. A propensity score analysis was used to compare the difference between the two triple therapy regimens. A propensity score analysis was also carried out to control for selection biases and compared effects between AC and ACCi or between AC and ACSa on incidence of cardiac or cerebral events. Propensity scores were calculated using a multivariate logistic regression analysis. After the matching of each group, we performed a Cox proportional hazard analysis to evaluate the effect of dual and triple therapies on the incidence of MACCE. The analysis was adjusted for the following baseline covariates: age, sex, comorbidities, and concomitant medications. Outcomes were presented as hazard ratios and 95% confidence intervals. The proportional hazard assumption was checked by examining the log-log plots of the hazard functions for each group. All of the statistical analyses were performed using SAS version 9.4 (SAS Institute, Cary, NC, USA)

### Ethical Approval

The study obtained an official approval from the HIRA inquiry commission. Each patient’s personal privacy was protected by de-identification of the national insurance claim data for analysis.

## Results

Total patients of the study cohort were 4,341,608 with a diagnosis of ischemic heart disease between January 1, 2009 and December 31, 2013. Out of 93,876 patient with a stent procedure identified in the cohort, 69,491 patients were included in the study who were administered with the antiplatelet therapy of aspirin, clopidogrel, cilostazol or sarpogrelate as the study drug regimens. Dual therapy with aspirin and clopidogrel (AC) was administered in 50,117 (72.1%); triple therapy aspirin, clopidogrel, and cilostazol (ACCi) in 18,002(25.9%); and aspirin, clopidogrel, and sarpogrelate (ACSa) in 1,372 (2.0%) patients (Fig 1).

Baseline characteristics, comorbidity and concomitant medications are reported in Table 1. The triple therapy ACCi and ACSa groups had not only higher Charlson comorbidity scores, but also higher rates of hypertension, diabetes, peripheral atrial diseases, and previous PCI compared with the AC group. The ACSa group was older than AC and ACCi groups (ACSa: 65.7 ± 10.1, ACCi: 63.8 ± 10.9, AC: 63.6 ± 6 years), and had a higher percentage of females compared to the other groups (ACSa: 40.5%, ACCi: 30.2%, AC: 31.9%,). The ACSa group exhibited a higher rate of treatment of more than two vessels compared with other groups (ACSa: 11.8%, ACCi: 6.4%, AC: 6.2%). The mean time of duration until the censored event was 273± 151days.

**Table 1:** Baseline characteristics of the study population.

	Overall population (n = 69,491)				
	ACSa	ACCi	AC	SMD	SMD
	(n = 1,372)	(n = 18,002)	(n = 50,117)	(ACSa)	(ACCi)
**Age, years**					
Mean ± SD	65.7 ± 10.1	63.8 ± 10.9	63.6 ± 6.0	0.34	0.02
**Age, n (%)**					
Less than 65 years	575 (41.9)	8,922 (49.6)	25,481 (50.8)	0.006	0.029
65–74 years	510 (37.2)	6,081 (33.8)	15,680 (31.3)		
75 years and older	287 (20.9)	2,999 (16.7)	8,956 (17.9)		
**Male, n (%)**	816 (59.5)	12,559 (69.8)	34,114 (68.1)	0.054	0.059
**Charlson Comorbidities**	3.00 ± 1.7	2.71 ± 1.6	2.19 ± 1.6	0.329	0.15
**Index score (mean+/-SD)**					
**Comorbidities, n (%)**					
Hyperlipidemia	1,089 (79.4)	14,576 (80.9)	39,353 (78.5)	0.005	0.007
Hypertension	1,150 (83.8)	14,802 (82.2)	38,638 (77.1)	0.016	0.009
Type II diabetes mellitus	746 (54.4)	9,302 (51.7)	19,117 (38.1)	0.002	0.004
Cerebrovascular disease	273 (19.9)	3,139 (17.4)	6,563 (13.1)	0.003	0.032
MI	318 (23.2)	5,903 (32.8)	15,404 (30.7)	0.014	0.016
CRD (stage 1–3)	5 (0.36)	20 (0.11)	69 (0.14)	0.007	0.005
PAD	240 (17.5)	2,327 (12.9)	4,312 (8.6)	0.004	0.042
Coagulopathy	10 (0.73)	54 (0.30)	165 (0.32)	0.009	0.004
AF	54 (3.96)	572 (3.18)	1564 (3.12)	0.012	0.006
Previous PCI	94 (6.9)	1,147 (6.4)	1,866 (3.7)	0.016	0.049
Previous CABG	2 (0.15)	33 (0.18)	87 (0.17)	0.011	0.001
**Clinical diagnosis, n (%)**					
Stable angina	304 (22.2)	4,263 (23.7)	12,857 (25.7)	0.067	0.038
Unstable angina	576 (42.0)	6,249 (34.7)	16,109 (32.1)	0.084	0.054
Silent ischemia	5 (0.36)	58 (0.32)	213 (0.43)	0.013	0.022
MI	287 (20.1)	5,297 (29.4)	14,286 (28.5)	0.009	0.014
Unknown	200 (14.6)	2,135 (11.9)	6,652 (13.3)	0.015	0.041
1 vessel	1,284 (93.6)	15.873 (88.2)	47,012 (93.8)	0.006	0.102
>1 vessel	88 (6.4)	2,129 (11.8)	3,105 (6.2)		
**Concomitant medications, n (%)**					
ACEIs/ARBs	759 (55.3)	10,451 (58.1)	27,273 (54.4)	0.008	0.012
Nitrates	132 (9.6)	2,028 (11.3)	4,837 (9.7)	0.019	0.038
BB	711 (51.8)	10,401 (57.8)	27,392 (54.7)	0.002	0.018
CCB	672 (49.0)	6,942 (38.6)	19,662 (39.2)	0.066	0.043
Statins	1,064 (77.6)	14,951 (83.1)	41,386 (82.6)	0.052	0.008
Other lipid lowering agents	115 (8.4)	1,215 (6.8)	3,976 (7.9)	0.007	0.049
PPI	65 (4.7)	1,005 (5.6)	2,088 (4.2)	0.002	0.057

AC, aspirin+clopidogrel; ACCi, aspirin+clopidogrel+cilostazol; ACSa, aspirin+clopidogrel+sarpogrelate

MI, myocardial infarction; CRD, Chronic renal disease; PAD, peripheral arterial disease; AF, atrial fibrillation; PCI, percutaneous coronary intervention; CABG, coronary artery bypass graft surgery; ACEI, angiotensin converting enzyme inhibitor; ARB, angiotensin receptor blocker; BB, beta blocker; CCB, calcium channel blocker; PPI, proton pump inhibitor; SMD (ACSa), standardized mean difference between AC and ACSa groups after the propensity score matching (10:1); SMD (ACCi), standardized mean difference between AC and ACCi groups after the propensity score matching (3:1)

**Fig 2:**
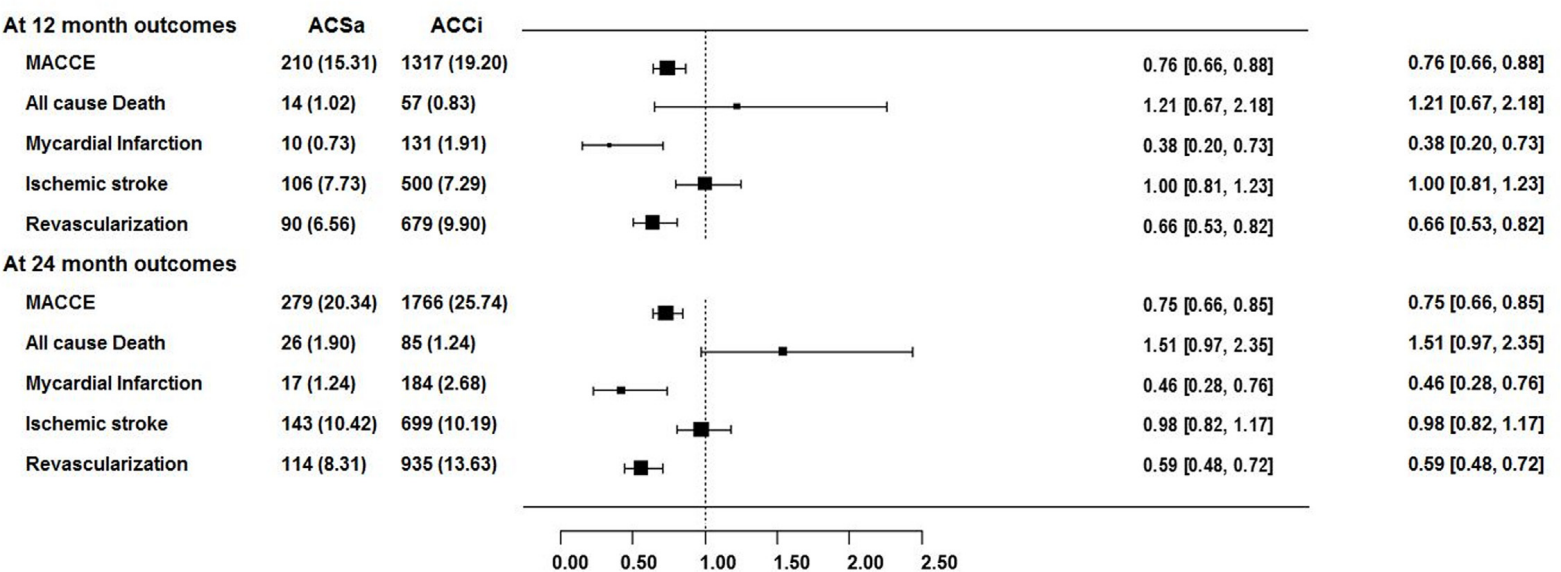
Clinical outcomes of incidence rates and relative risks of cardiac events between ACCi and ACSa. ACCi, aspirin+clopidogrel+cilostazol; ACSa, aspirin+clopidogrel+sarpogrelate; MACCE, major adverse cardiac cerebral events (composite events of death, myocardial infarction, ischemic stroke, revascularization).

The clinical outcomes of ACSa therapy showed the beneficial effects in the prevention of subsequent cardiac or cerebral events. After propensity score matching with age, gender and comorbidity between ACSa and ACCi groups, 2,045 (24.8%) patients out of total 8,232 developed MACCE including 111 all cause death, 201 MI, 842 ischemic stroke and 1,049 revascularization at 24 months after PCI. There were significant differences in incidents of MI and revascularization, with corresponding HR of 0.38 (95% CI, 0.20–0.73) and 0.66 (95% CI, 0.53–0.82) in the ACSa vs. ACCi at 12 months after PCI, respectively (Table 2 and Fig 2).

**Table 2:** Clinical outcomes of Incidence rates and relative risks of cardiac or cerebral events.

		ACSa	ACCi	AC	ACSa vs. ACCi	ACSa vs. AC	ACCi vs. AC
At 12 month		n = 1,372	n = 18,002	n = 50,117	Propensity score-adjusted HR (95% CI) ^†^, p-value		
**MACCE**	Patients with events (n)	210	3,489	5,402	0.76 (0.66–0.88)	1.22 (1.06–1.41)	1.69 (1.62–1.77)
	Patient-years (PYs)	499,196	6,244,739	18,601,499	p <0.001	p = 0.01	p <0.001
	Incidence rate per 100 PYs (IR/100 PYS)	4.2	5.6	2.9			
**All-cause death**	Patients with events (n)	14	165	485	1.21 (0.67–2.17)	1.01 (0.59–1.76)	0.89 (0.74–1.07)
	PYs	547,572	7,171,289	19,954,125	p = 0.53	p = 0.96	p = 0.21
	IR/100 PYS	0.3	0.2	0.2			
**Myocardial Infarction**	Patients with events (n)	10	360	380	0.38 (0.20–0.73)	1.04 (0.54–1.99)	2.55 (2.18–2.98)
	PYs	544,861	7,081,427	19,919,603	p = 0.01	p = 0.92	p <0.001
	IR/100 PYS	0.2	0.5	0.2			
**Ischemic Stroke**	Patients with events (n)	106	1204	2300	1.00 (0.81–1.23)	1.19 (0.97–1.46)	1.19 (1.10–1.27)
	PYs	520,979	6,856,165	19,378,555	p = 0.99	p = 0.08	p <0.001
	IR/100 PYS	2.0	1.8	1.8			
**Revascularization**	Patients with events (n)	93	1934	2432	0.66 (0.53–0.82)	1.38 (1.11–1.72)	2.24 (2.10–2.39)
**(PCI+CABG)**	PYs	533,791	6,879,861	19,662,565	p < 0.01	p = 0.01	p <0.001
	IR/100 PYS	1.7	2.8	1.2			
**At 24 month**							
**MACCE**	Patients with events (n)	279	4,636	7,223	0.75 (0.66–0.85)	1.24 (1.09–1.40)	1.72 (1.65–1.79)
	PYs	948,444	11,744,441	36,053,497	p <0.001	p = 0.00	p <0.001
	IR/100 PYS	2.9	3.9	2.0			
**All-cause death**	Patients with events (n)	26	236	722	1.51 (0.97–2.34)	1.19 (0.79–1.78)	0.84 (0.72–0.97)
	PYs	1,090,608	14,087,477	39,788,881	p = 0.07	p = 0.41	p = 0.02
	IR/100 PYS	0.2	0.2	0.2			
**Myocardial Infarction**	Patients with events (n)	17	534	531	0.46 (0.28–0.76)	1.13 (0.69–1.87)	2.69 (2.37–3.07)
	PYs	1,087,832	14,087,477	39,769,609	p = 0.01	p = 0.63	p <0.001
	IR/100 PYS	0.2	0.4	0.1			
**Ischemic Stroke**	PYs	143	1,673	3,114	0.98 (0.82–1.17)	1.26 (1.06–1.49)	1.27 (1.19–1.35)
	IR/100 PYS	1,019,428	13,476,971	38,334,927	p = 0.83	p = 0.01	p <0.001
	Incidence rate per 100 PYs	1.4	1.2	0.8			
**Revascularization**	Patients with events (n)	114	2,539	3,230	0.59 (0.49–0.72)	1.26 (1.04–1.54)	2.22 (2.10–2.35)
**(PCI+CABG)**	PYs	1,039,675	13,152,732	38,530,830	p <0.001	p = 0.02	p <0.001
	IR/100 PYS	1.1	1.9	0.8			

MACCE, Major adverse cardiac or cerebral events; MI, Myocardial Infarction; AC, aspirin + clopidogrel; ACSa, aspirin + clopidogrel + sarpogrelate; ACCi, aspirin + clopidogrel + cilostazol

^**†**^PS Matching (ACCi:ACSa = 5:1 matching, AC:ACCi = 3:1 matching, AC:ACSa = 10:1 matching)

Clinical outcomes of triple antiplatelet therapies, ACSa and ACCi, compared to a dual antiplatelet therapy, AC are shown in Table 2. During the follow-up period, 12,138 (17.5%) out of 69,491 patients developed MACCE, including 984 all-cause death,1,028 MI, 4,930 ischemic strokes, and 5,883 revascularizations. The HR of death, MI, and ischemic stroke for the ACCi group compared with the AC group were 0.89 (95% CI, 0.74–1.07), 2.55 (95% CI, 2.18–2.98), and 1.043 (95% CI, 1.10–1.27), respectively. The HR of revascularization for the ACCi group compared with the AC group was 2.24 (95% CI, 2.10–2.39). When potential confounding factors were adjusted for, the risk of the ACSa groups compared with the AC group had no difference for all cardiac adverse events except revascularization. The HR of death, MI, ischemic stroke, and revascularization at 12 months for the ACSa group compared to the AC group were 1.01 (95% CI, 0.59–1.76), 1.04 (95% CI, 0.54–1.99), 1.19 (95% CI, 0.97–1.46), and 1.38 (95% CI, 1.11–1.72), respectively. As shown in Table 3, there was no significant difference in severe or life-threatening bleeding risk among three groups. Multivariable Cox regression analysis of severe or life-threatening bleeding showed that the ACCi and ACSa groups had no significant increase in risk compared to the AC group; ACCi vs. AC, HR 0.91 (95% CI, 0.77–1.09) and ACSa vs. AC, HR 0.68 (95% CI, 0.37–1.24).

**Table 3:** Safety outcomes of incidence rates and relative risks of bleeding complications.

	Antiplatelet therapies, total (n = 69,491)						
	ACSa	ACCi	AC	Adjusted HR(95% CI)	p-value	Adjusted HR (95% CI)	p-value
	(n = 1,372)	(n = 18,002)	(n = 50,117)	ACSa vs. AC		ACCi vs. AC	
Severe or life-threatening bleeding	11 (0.80)	178 (0.99)	462 (0.92)	0.68 (0.38–1.24)	0.78	0.91 (0.77–1.09)	0.91
Moderate or mild bleeding	174 (12.68)	1,804 (10.02)	4,639 (9.26)	1.31 (1.13–1.53)	0.01	1.04 (0.99–1.10)	0.21

AC, aspirin+clopidogrel; ACCi, aspirin+clopidogrel+cilostazol; ACSa, aspirin+clopidogrel+sarpogrelate

## Discussion

The prevention of MACCE is a key clinical goal in patients with stent procedure of PCI. Triple therapy with cilostazol or sarpogrelate has been used for patients with additional needs, such as diabetes or other comorbidities. In this study, we found that sarpogrelate showed a clinical outcomes as antiplatelet drugs that can be added to dual antiplatelet therapy (aspirin and clopidogrel) in terms of prevention of cardiac or cerebral events in patients with a stent procedure. Dual antiplatelet therapy (aspirin and clopidogrel) have been widely used in patients undergoing PCI. However, several studies have reported that up to 30% of patients with cardiovascular disease are resistant to clopidogrel, and 10% of patients undergoing PCI exhibit poor outcomes when taking aspirin and clopidogrel [25, 26]. Studies have confirmed that clopidogrel resistance is explained by inter-individual differences in genetic polymorphism of CYP2C19 expression [7]. The variant CYP2C19 allele causes decreased formation of the active metabolite of clopidogrel, and thereby reduces efficacy of clopidogrel in inhibiting ADP-induced platelet activation [27]. Platelet reactivity on clopidogrel detected by P2Y12 assay was associated with increased long-term cardiovascular events after PCI in a meta-analysis [28].

Increasing the dose of clopidogrel had been suggested as a measure to overcome resistance; however, the GRAVITAS trial showed that there was no added benefit from a high dose of clopidogrel [29]. Maruyama H. showed that adding cilostazol to aspirin and clopidogrel can attenuate the clopidogrel resistance [30, 31]. Shim et al. also reported that triple therapy with cilostazol was more effective than dual therapy in overcoming clopidogrel resistance in patients undergoing PCI [32]. The efficacy of the triple combination using cilostazol has been studied in several randomized controlled trials in various populations. Lee et al. reported the efficacy of a triple combination using cilostazol after stenting in the DECLARE-DIABETES and DECLARE-LONG trial [10, 11, 33]. Chen et al. and Han et al. also demonstrated the efficicay of triple therapies with cilostazol [8, 9]. Other P2Y12-receptor antagonists, such as prasugrel and ticagrelor were recommended instead of clopidogrel, especially to high-risk patients in clinical practice guidelines [1, 2]. However, the guidelines have been developed by published data based on European or American population rather than Asian. Based on several RCT trials conducted with Asian population, Korean guidelines of antiplatelet therapy recommends cilostazol with aspirin and clopidogrel to reduce clopidogrel resistance [14]. Cilostazol and sarpogrelate were preferred in patients with diabetes or peripheral vascular disease and have been added as a third agent instead of using prasugrel and ticagrelor in place of clopidogrel in Korea. In addition, the FDA and EMA recently approved vorapaxar in addition to aspirin and clopidogrel to reduce ischemic events in patients with MI [34]. Vorapaxar, an antagonist of the protease-activated receptor-1 (PAR-1) expressed on platelets, inhibits platelet aggregation irreversibly [35]. It is used once orally in combination with aspirin and/or clopidogrel with contraindication in patients with history of stroke, TIA, or ICH [36]. There were no experience with use of vorapaxar as monotherapy or with antiplatelet agents other than aspirin and clopidogrel. Vorapaxar was not approved in Korea yet and should be assessed in the future.

Sarpogrelate is considered as an alternative drug that can potentially prevent incidence of further cardiac events after undergoing PCI. It have been used as a platelet aggregation inhibitor to improve peripheral circulation in the treatment of ischemic symptoms observed in chronic arterial obstruction in South Korea [15]. Previous studies have reported on several aspects of sarpogrelate that indicate improved benefit in particular populations. First, vascular smooth muscle cell proliferation in response to vascular injury is mediated by 5-HT released from adhering platelets. Sarpogrelate inhibits vascular smooth muscle cell proliferation by direct activation 5-HT2A receptors, which functions to prevent restenosis. Fujita et al. demonstrated a significantly lower rate of restenosis after coronary stenting in patients with sarpogrelate added to the standard dual therapy [18]. In addition, sarpogrelate may also be beneficial in treating ischemia by inhibiting 5-HT levels in the heart and reducing infarct size. The S-ACCESS trial showed that sarpogrelate was more effective in preventing recurrence in patients with recent ischemic stroke compared to aspirin [37].

A national insurance claims data was used to include enough number of patients in the retrospective study from the real-world clinical practice. For comparing efficacy between two triple therapies, ACSa and ACCi groups were similar to each other in terms of baseline patient characteristics with propensity score matching. As a results, ACSa group had a lower incidence of revascularization compared to ACCi group, as well as a much lower incidence of MI. Sarpogrelate might help to reduce the platelet adhesion and aggregation by inhibition of 5-HT2 receptors, because serotonin increased residual platelet reactivity [38].

In this retrospective cohort study, both triple therapy groups (ACSa and ACCi) had much higher comorbidity of hypertension, DM, peripheral arterial disease (PAD), and cerebrovascular disease (CVD) at baseline compared to the AC group. Therefore, each triple therapy groups also were matched with AC group with propensity score matching for comparing impact between the triple therapy and the dual therapy. The risk of incidence of MACCE in triple therapy groups was shown to be higher than the AC group. A significant difference in revascularization incidence was shown in the ACCi group compared with the AC group. In the DECLARE randomized trials, adding cilostazol to the standard care conveyed significant reductions in TLR and angiographic restenosis. In other RCT studies, however, there was no difference in revascularization between a triple therapy group and the standard dual therapy [8, 9]. In the observation study, there was also no difference for reduction of revascularization between two therapies [39]. The possible reason for this discrepancy could be a difference in type of stents used or lesions treated. In our data, ACCi group was shown to much higher number of treated mutiple vessels, which might influence to the results. While the difference in baseline characteristics was adjusted statistically, this adjustment could not comletely correct for the uneven distribution of unobservable factors, such as stent types.

There was no statistically significant difference in comparing incidence of other cardiac or cerebral events in ischemic stroke and all-cause death among three groups. Han et al. and Song et al. also reported no difference in preventing ischemic stroke in groups taking triple antiplatelet therapy [8, 39]. In our studies, increase in bleeding risk in the ACCi and ACSa groups was not reported in this study. Although minor bleeding risk in ACSa groups was a little higher than other groups, there was no statistically significant difference. In contrast, the severe bleeding risk in ACSa group was shown relatively lower than others. Previous studies reported that the bleeding risk of the triple antiplatelet was not significantly high [5, 8–10, 40]. Our results of bleeding based on GUSTO were consistent with previous studies. The criteria of bleeding of ISTH or TIMI could not been applied in this study due to the limitation of database which did not provide the hemoglobin level or the amount of blood transfusion. The finding of this study must be interpreted with caution, there is still concern that concomitant use of multiple antiplatelet agents could increase the bleeding risk. Even though the data did not show the significantly higher bleeding risk statistically, the monitoring of bleeding would be necessary with triple antipletelet therapy.

In this study, cilostazol in combination with standard dual antiplatelet therapy did not improve significantly efficacy in preventing MACCE, but sarpogrelate in combination with standard therapy was more effective to the cilostazol. Several pharmacological advantages of sarpogrelate make it a good candidate for antiplatelet therapy after PCI with stent procedures. It has a relatively short half-life and is reversible, therefore it can be used if a short withdrawal period is desired. Furthermore, adverse effects due to vasodilation are relatively low compared to prostaglandin agonists or cilostazol, which may improve adherence to therapy.

Use of the national health insurance claims database for a retrospective analysis was a strength of the study, since the results could represent the general population of Korea, or about 50 million people. Follow-up data of most patients was complete, because the entire claim data of the patient was been collected in the database, regardless of the setting in which they received care.

### Limitation

Our study also had some limitations. Because this study was an observational study using insurance administration data, certain clinical information that may have influenced the outcome were not included, such as family history, tobacco use, or BMI. Second, the data were isolated on the basis of ICD-10 diagnosis codes, and coding errors may have led to under- or over-estimation of the outcomes. However, previous studies have reported the accuracy of ICD-10 codes in myocardial infarction and cerebrovascular disease, which is correct more than 70% of the time for MI and 83.4% for ischemic stroke [41]. Third, the number of patients in sarpogrelate is small since it is not included in the PCI guideline and not the reimbursement of insurance. The efficacy of sarpogrelate and cilostazol is a high interest in patients with additional risk factors of atherosclerosis. Sarpogrelate was considered as alternative addition of triple therapy in Korea. The clinical investigation, SERENADE is in progress for prevention of MACCE after PCI in patients with renal impairment or diabetes [42]. The efficacy and safety of sarpogrelate need more evidence compared to the existing treatment with real world data and controlled clinical trial. We tried to analyze the practical data although the difference number of population in three groups were significant. The characteristics of three groups was adjusted by propensity score matching to reduce the bias from the difference among groups. Fourth, the number of vessels were identified as one or multiple vessels in the procedure code of the claim data, but the type of stents or location of treated vessels could not be identified. The genetic testing for clopidogrel low response has not been carried out and reimbursed yet in Korea. The information of the genetic testing was not available in our claim data. Fifth, sarpogrelate has been studied only in the Asian population. To verify the efficacy of sarpogrelate in a global population, a large randomized study including patients from other ethnicities is also needed.

In conclusion, our study suggests that using sarpogrelate as an adjunct to conventional dual antiplatelet therapy after PCI could be of benefit in certain populations such as patients who experienced treatment failure due to clopidogrel resistance or high risk patients, and it showed comparable rates of clinical outcomes without significantly increasing bleeding risk over a two-year follow-up period.

## Supporting Information

S1 Table Defined ICD-10 codes. (DOCX)

S2 Table Baseline characteristics of ACS vs. ACC after PS Matching. (DOCX)
