# Supplementary material for: Antiplatelet Therapy of Cilostazol or Sarpogrelate with Aspirin and Clopidogrel after Percutaneous Coronary Intervention: A Retrospective Cohort Study Using the Korean National Health Insurance Claim Database
Source: PLoS One. 2016 Mar 3;11(3):e0150475. doi: 10.1371/journal.pone.0150475 (PMC4777511; doi:10.1371/journal.pone.0150475)
Supplement: S1 Table — (DOCX) [file pone.0150475.s001.docx]

**Supporting Information**

**S1 Table. Defined ICD-10 codes**

| MI | I21 | Acute myocardial infarction |
| --- | --- | --- |
|  | I22 | Subsequent myocardial infarction |
| Ischemic stroke | I63 | Cerebral infarction |
|  | I64 | Stroke, not specified as hemorrhage or infarction |
| death | I46 | Cardiac arrest |
|  | R96 | Other sudden death, cause unknown |
|  | R98 | Unattended death |
|  | R99 | Other ill-defined and unspecified causes of mortality |
| Severe or life-threatening bleeding | I60 | Subarachnoid hemorrhage |
|  | I61 | Intracerebral hemorrhage |
|  | I62 | Other nontraumatic intracranial hemorrhage |
| Moderate and mild bleeding | H05.2 | Haemorrhage of orbit |
|  | H11.3 | Conjunctival hemorrhage |
|  | H11.3 | Subonjunctival harmorrhage |
|  | H21.0 | Hyphaema |
|  | H31.3 | Choroidal hemorrhage and rupture |
|  | H31.3 | Choroidal haemorrage NOS |
|  | H31.3 | Explusive choroidal haemorrhage |
|  | H35.6 | Retinal hemorrhage |
|  | H43.1 | Vitreous hemorrhage |
|  | H47.0 | Haemorrhage in optic nerve sheath |
|  | I84.10 | Bleeding internal hemorhoids |
|  | I84.40 | Bleeding external hemorrhoids |
|  | I84.80 | Unspecified bleeding haemorrhoids |
|  | K22.11 | Ulcer of oesophagus with bleeding |
|  | K22.8 | Hemorrhage of oesophagus NOS |
|  | K25.0 | Acute gastric ulcer with hemorrhage |
|  | K25.2 | Acute gastric ulcer with both hemorrhage and perforation |
|  | K26.0 | Acute duodenal ulcer with hemorrhage |
|  | K26.2 | Acute duodenal ulcer with both hemorrhage and perforation |
|  | K27.0 | Acute peptic ulcer, site unspecified with hemorrhage |
|  | K27.2 | Acute peptic ulcer, site unspecified with both hemorrhage and perforation |
|  | K28.0 | Acute gastrojejunal ulcer with hemorrhage |
|  | K28.2 | Acute gastrojejunal ulcer with both hemorrhage and perforation |
|  | K29.0 | Acute hemorrhagic gastritis |
|  | K57.01 | Diverticular disease of small intestine with perforation and abscess, Diverticulosis, with bleeding |
|  | K57.03 | Diverticular disease of small intestine with perforation and abscess, Diverticulitis, with bleeding |
|  | K57.11 | Diverticular disease of small intestine without perforation or abscess, Diverticulosis, with bleeding |
|  | K57.13 | Diverticular disease of small intestine without perforation or abscess, Diverticulitis, with bleeding |
|  | K57.21 | Diverticular disease of large intestine with perforation and abscess, Diverticulosis, with bleeding |
|  | K57.23 | Diverticular disease of large intestine with perforation and abscess, Diverticulitis, with bleeding |
|  | K57.31 | Diverticular disease of large intestine without perforation or abscess, Diverticulosis, with bleeding |
|  | K57.33 | Diverticular disease of large intestine without perforation or abscess, Diverticulitis, with bleeding |
|  | K57.41 | Diverticular disease of both samll and large intestine with perforation and abscess, Diverticulosis, with bleeding |
|  | K57.43 | Diverticular disease of both samll and large intestine with perforation and abscess, Diverticulitis, with bleeding |
|  | K57.51 | Diverticular disease of both small and large intestine without perforation or abscess, Diverticulosis, with bleeding |
|  | K57.53 | Diverticular disease of both small and large intestine without perforation or abscess, Diverticulitis, with bleeding |
|  | K57.81 | Diverticular disease of intestine, part unspecified, with perforation and abscess, Diverticulosis, with bleeding |
|  | K57.83 | Diverticular disease of intestine, part unspecified, with perforation and abscess, Diverticulitis, with bleeding |
|  | K57.91 | Diverticular disease of intestine, part unspecified, without perforation or abscess, Diverticulosis, with bleeding |
|  | K57.93 | Diverticular disease of intestine, part unspecified, without perforation or abscess, Diverticulitis, with bleeding |
|  | K62.5 | Hemorrhage of anus and rectum |
|  | K85.8 | Haemorrhagic pancreatitis |
|  | K92.2 | Gastric hemorrhage NOS |
|  | K92.2 | Intestinal hemorrhage NOS |
|  | N50.1 | Hemorrhage of male genital organs |
|  | N83.0 | Hemorrhagic follicular cyst (of ovary) |
|  | N83.1 | Hemorrhagic corpus luteum cyst |
|  | R04 | Hemorrhage from respiratory passages |
|  | R58 | Hemorrhage, NEC |
| Stable angina | I20.9 | Angina pectoris |
| Silent ischemia | I25.6 | Silent myocardial ischemia |
| Unstable angina | I20.0 | Unstable angina |
| PAD | I73 | Other peripheral vascular diseases |
| Hypertension | I10 | Essential(primary) hypertension |
|  | I11 | Hypertensive heart disease |
|  | I12 | Hypertensive renal disease |
|  | I13 | Hypertensive heart and renal disease |
|  | I15 | Secondary hypertension |
| Diabetes | E11 | Non-insulin-dependent diabetes mellitus |
|  | E12 | Malnutrition-related diabetes mellitus |
|  | E13 | Other specified diabetes mellitus |
|  | E14 | Unspecified diabetes mellitus |
| Cerebrovascular | I60 | Subarachnoid hemorrhage |
|  | I61 | Intracerebral hemorrhage |
|  | I62 | Other nontraumatic intracranial hemorrhage |
|  | I63 | Cerebral infarction |
|  | I64 | Stroke, not specified as hemorrhage or infarction |
|  | I65 | Occlusion and stenosis of precerebral arteries, not resulting in cerebral infarction |
|  | I66 | Occlusion and stenosis of cerebral arteries, not resulting in cerebral infarction |
|  | I67 | Other cerebrovascular diseases |
|  | I68 | Cerebrovascular disorders in diseases classified elsewhere |
|  | I69 | Sequelae of cerebrovascular disease |
| Hyperlipidemia | E78 | Disorders of lipoprotein metabolism and other lipidemias |
| CRD | N18.1 | Chronic kidney disease, stage 1 |
|  | N18.2 | Chronic kidney disease, stage 2 |
|  | N18.3 | Chronic kidney disease, stage 3 |
