# Supplementary material for: Antiplatelet Therapy of Cilostazol or Sarpogrelate with Aspirin and Clopidogrel after Percutaneous Coronary Intervention: A Retrospective Cohort Study Using the Korean National Health Insurance Claim Database
Source: PLoS One. 2016 Mar 3;11(3):e0150475. doi: 10.1371/journal.pone.0150475 (PMC4777511; doi:10.1371/journal.pone.0150475)
Supplement: S2 Table — (DOCX) [file pone.0150475.s002.docx]

**Supporting Information**

**S2 Table. Baseline characteristics of ACS vs. ACC after PS Matching**

|  | **ACS** | **ACC** | **P-value** |
| --- | --- | --- | --- |
|  | **(n = 1,372)** | **(n = 6,860)** |  |
| **Age, years** |  |  |  |
| Mean ± SD | 65.7±10.1 | 65.3±10.5 | 0.23 |
| **Age, n (%)** |  |  |  |
| Less than 65 years | 575 (41.9) | 2,900 (42.3) | 0.9 |
| 65-74 years | 510 (37.2) | 2,563 (37.4) |  |
| 75 years and older | 287 (20.9) | 1,397 (20.3) |  |
| **Male, n (%)** | 816 (59.5) | 4,616 (67.3) | <0.0001 |
| **Charlson Comorbidities** | 3.0 ±1.7 | 2.8 ±1.7 | <0.0001 |
| **Index score (mean+/-SD)** |  |  |  |
| **Comorbidities, n (%)** |  |  |  |
| Hyperlipidemia | 1,089 (79.4) | 5,477 (79.8) | 0.69 |
| Hypertension | 1,150 (83.8) | 5,772 (84.1) | 0.77 |
| Type II diabetes mellitus | 746 (54.4) | 3,687 (53.8) | 0.67 |
| Cerebrovascular disease | 273 (19.9) | 1,319 (19.2) | 0.57 |
| MI | 318 (23.2) | 1,532 (22.3) | 0.49 |
| CRD (stage 1-3) | 5 (0.36) | 18 (0.26) | 0.51 |
| PAD | 240 (17.5) | 1,182 (17.2) | 0.81 |
| Previous PCI | 94 (6.8) | 382 (5.6) | 0.06 |
| Previous CABG | 2 (0.15) | 16 (0.2) | 0.75 |
| **Clinical diagnosis, n (%)** |  |  |  |
| Stable angina | 304 (22.2) | 1,826 (26.6) | 0 |
| Unstable angina | 576 (41.9) | 2,631 (38.4) | 0.01 |
| Silent ischemia | 5 (0.4) | 20 (0.3) | 0.65 |
| MI | 287 (20.9) | 1,493 (21.8) | 0.49 |
| Unknown | 200 (14.6) | 890 (13.0) | 0.11 |
| 1 vessel | 1,248 (93.6) | 6,011 (87.6) | <0.0001 |
| >1 vessel | 88 (6.4) | 849 (12.4) |  |
| **Concomitant medications, n (%)** |  |  |  |
| ACEIs/ARBs | 759 (55.3) | 3,774 (55.0) | 0.84 |
| Nitrates | 132 (9.6) | 797 (9.7) | 0.03 |
| BB | 711 (51.8) | 3,510 (51.2) | 0.66 |
| CCB | 672 (49.0) | 2,846 (41.5) | <0.0001 |
| Statins | 1,064 (77.6) | 5,610 (81.8) | 0 |
| Other lipid lowering agents | 115 (8.4) | 426 (6.2) | 0 |
| PPI | 65 (4.7) | 407 (5.9) | 0.08 |
